# Examining differential success in recruitment using respondent driven sampling (RDS) in a multi-site study of gay, bisexual and other men who have sex with men

**DOI:** 10.1186/s12874-023-01886-9

**Published:** 2023-06-09

**Authors:** Jordan M. Sang, Bita Gholamian, Lu Wang, Justin Barath, Syed W. Noor, Nathan J. Lachowsky, Trevor A. Hart, Joseph Cox, Gilles Lambert, Daniel Grace, Shayna Skakoon-Sparling, Allan Lal, Abbie Parlette, Herak Apelian, Jody Jollimore, Robert S. Hogg, David M. Moore

**Affiliations:** 1 British Columbia Centre for Excellence in HIV/AIDS, Vancouver, BC Canada; 2 Simon Fraser University, Burnaby, BC Canada; 3 Ryerson University, Toronto, ON Canada; 4 School of Human Sciences, Louisiana State University Shreveport, Shreveport, USA; 5 University of Victoria, Victoria, BC Canada; 6 Community Based Research Centre, Vancouver, BC Canada; 7 University of Toronto, Toronto, ON Canada; 8 Research Institute of the McGill University Health Center, Montréal, QC Canada; 9 Direction régionale de santé publique -Montréal, CIUSSS Centre-Sud-de-l’Ile-de-Montréal, Montréal, QC Canada; 10 Institut national de santé publique du Québec, Montréal, QC Canada; 11 University of British Columbia, Vancouver, BC Canada

**Keywords:** Respondent-driven sampling, GBM, Multi-site studies, Methodology

## Abstract

**Background:**

The Engage Study is a longitudinal biobehavioral cohort study of gay, bisexual and other men who have sex with men (GBM) in Toronto, Montreal, and Vancouver. Baseline data (2,449 participants) were collected from February 2017 - August 2019 using respondent-driven sampling (RDS). Recruitment in Montreal required fewer seeds, had a much shorter recruitment period, and recruited the largest sample.

**Methods:**

To better understand why RDS recruitment was more successful in Montreal compared to other sites, we conducted an analysis to examine RDS recruitment characteristics for GBM in each of the three study sites, explore demographic characteristics and measures of homophily, that is, the tendency of individuals to recruit other study participants who are like themselves, and compared motivations for study participation.

**Results:**

Montreal had the greatest proportion of participants over the age of 45 (29.1% in Montreal, 24.6% in Vancouver, and 21.0% in Toronto) and the highest homophily for this age group, but homophily was high across the three cities. Montreal also reported the lowest percentage of participants with an annual income greater or equal to $60,000 (7.9% in Montreal, 13.1% in Vancouver and 10.6% in Toronto), but homophily was similar across all three cities. The majority of participants indicated interest in sexual health and HIV as the main reason for participating (36.1% in Montreal, 34.7% in Vancouver, and 29.8% in Toronto). Financial interest as the main reason for participation was low (12.7% in Montreal, 10.6% in Vancouver, and 5.7% in Toronto).

**Conclusion:**

Taken together, although we found some differences in study demographic characteristics and homophily scores, we were unable to fully explain the different recruitment success based on the data available. Our study underlines the fact that success of RDS implementation may vary by unknown factors, and that researchers should be proactive and flexible to account for variability.

## Introduction

Gay, bisexual, and other men who have sex with men (GBM) are disproportionately affected by HIV in Canada, with 41.4% of new diagnoses attributed to adult GBM in 2018 [1]. Ontario, Quebec, and British Columbia are three of the four provinces (the fourth being Alberta) with the highest number and proportion of new reported HIV cases, which include the country’s largest metropolitan areas of Toronto, Montreal, and Vancouver [1]. The disproportionate HIV burden among GBM has generated numerous studies to examine disease spread, as well as inform prevention and care interventions. A variety of methods have been used to recruit GBM into research studies, including time-location sampling [2, 3], clinical cohorts, [4] and convenience sampling [5]. In recent years, respondent-driven sampling (RDS), a form of peer referral-based sampling strategy, has increased in popularity as a method to recruit representative samples from hidden populations when a sampling frame is not available, including GBM [6, 7].

The Engage Study is a cohort study of GBM in Canada’s three largest cities (Vancouver, Toronto, and Montreal) [8]. The study recruited participants between February 2017 and August 2019 using RDS. Each site followed the same recruitment protocol with a target sample size of N = 2,160 (n = 720 in each city). The Montreal site later received supplementary funds from the Québec government to increase their sample size to 1,200 participants. All sites began with 30 seed participants (herein, seeds) and monitored recruitment progress. Additional seeds were allowed to be added to maintain steady recruitment. After recruitment had ended, it was observed that Montreal had obtained the largest sample size (n = 1,179) over the shortest recruitment period across the three cities (493 days), while also using the smallest number of seeds and having the largest percentage of productive seeds (77.8%) [27]. In comparison, recruitment in Vancouver spanned the longest time period (895 days) and recruited 753 participants using 117 seeds, of which 60.7% were productive. Toronto used 96 seeds of which 53 (55.2%) were productive with a total recruited n of 517 participants over the second longest recruitment period (815 days) [8]. Based on RDS parameters, including number of seeds, number of productive seeds, final sample size and time to final recruitment, we found clear differences between the Montreal site and sites in Vancouver and Toronto. Thus, we sought to look for explanations within our data that may have impacted RDS success in the study sites.

For studies using RDS methodology, recruitment success in terms of sample size, recruitment time, and diversity of samples has varied. Where researchers have evaluated RDS methodology among GBM, many have linked sociodemographic characteristics and recruitment productivity [9–11]. Wirtz et al. (2021) evaluated the use of a modified RDS approach to explore RDS-related challenges when used as a recruitment strategy among young GBM. The authors found that targeted seed recruitment from clinical samples resulted in enrolled participants who reported stable housing, GBM who are living with HIV, and GBM who report PrEP use, while online seeds were more likely to report Latinx ethnicity [12]. The authors also utilized qualitative interviews and found small social network sizes, limited targeted recruitment referrals and experiences of marginalization, stigma and isolation were barriers to RDS recruitment among young GBM [12]. These findings build upon previous RDS analyses in Vancouver, which found that participants who reported a larger network size, positive HIV status, Indigenous identity, and sexual activity exclusively with males were more likely to recruit at least one study participant compared to those who did not recruit any [13]. Motivations for study participation have examined altruistic and financial factors for participation. Factors such as participant’s interest in research, perceptions on study importance, sense of gratification, and community and cultural related beliefs have been identified [14, 15]. Moreover, authors identified that the perceived potential success of studies can also influence motives and rates of study participation [16]. However, most research examining motivations for study participation among GBM have been in the context of clinical trials (specifically HIV vaccine trials) and less is known about motivations for participating in epidemiological cohort studies [14, 15]. Previous research using RDS methodology among GBM also points to the importance of homophily, which measures the extent of preferential recruitment within or outside of one’s own characteristic group [17]. Accounting for homophily helps to examine biases from recruiting like individuals on RDS point-estimates, and previous studies have shown homophily widely ranges by characteristic and population [18, 19]. Homophily also influences the number of waves to reach equilibrium (when the sample composition stabilizes and becomes independent of seeds), and higher homophily will require more waves to reach stability, resulting in greater coupon distribution, seed make-up, and final sample size [6, 20]. Examining the associations between referrers and participants, as well as reasons for participation, may further our understanding of how RDS recruitment can be most successful among GBM.

Using data from The Engage Study, we conducted an analysis to (1) examine RDS recruitment characteristics for GBM in each of the three study sites, with an emphasis on differences in the Montreal site compared with the other two sites, (2) examine homophily recruitment estimates in each of the three sites, including demographic characteristics, and (3) participant’s motivations for joining the study. Using these metrics, we aimed to analyze why RDS recruitment was more effective in Montreal compared to the other study sites.

## Methods

### Procedures

Eligibility criteria for The Engage Study included: being at least 16 years of age, identify as a man (including trans men), reporting sex with another man in the past six months, currently living in Vancouver, Toronto, or Montreal, and being able to complete the questionnaire in English or French (the latter only for participants in Montreal). Seeds were mainly recruited with the assistance of our Community Engagement Committees (CEC), comprising staff from local community-based organizations focused on GBM health promotion, including AIDS service organizations and diverse GBM community members. In Toronto and Vancouver, we also used advertisements on social networking applications, such as Grindr, Growlr, and Squirt, as well as posts on Facebook and Craigslist to raise awareness of the study and recruit potential seeds [8]. Of the 30 initial seeds selected in each city, at least 10 were men living with HIV, 10 self-identified as ethnic minorities, two self-identified as transgender, and two as bisexual. We also tried to recruit at least two seeds who were below the age of 18 years. All seeds and subsequent recruits were provided with six coupons to offer to other GBM from their social networks. Coupons were either provided as a printed card, or an electronic version which could be sent by text or email. Coupons had a unique identifier number to ensure that they could only be redeemed by one individual. Study sites monitored recruitment progress and new seeds were added as needed to promote recruitment representativity as pre-determined using community mapping. Additionally, participants were followed-up at structured timepoints (2–3 months after their initial visit) to inform them of any redeemed coupons and to encourage distribution of any unredeemed coupons. This follow-up was also used as an opportunity to address any participant questions or concerns. Full study procedures can be found elsewhere [8, 21, 22].

Participants completed a computer-assisted self-interview (CASI) which asked questions about sexual behaviours, sexual health, substance use, and demographics. Participants also completed a nursing visit where a study nurse performed screening for sexually transmitted infections (STIs) and HIV. For further information on the specific tests, please see Hart et al. (2021) [8]. For each visit, participants received an honorarium of $50 (CAD) (Equivalent to $40 USD) and an additional compensation of $15 CAD (Equivalent to $12 USD) for each eligible recruit who completed a study visit. All participants signed an informed consent form about their participation in the study. The study was approved by research ethics boards at Toronto Metropolitan University, University of Toronto, St. Michael’s Hospital, University of Windsor, University of British Columbia, Providence Health Care, University of Victoria, Simon Fraser University, and McGill University Health Centre.

### Measures

RDS process indicators included the date of first interview and the last interview date of participants, and the time difference in days between recruitment periods. We also included the number of seeds, number of productive seeds (i.e., seeds that produced at least one recruitment wave), the percentage of productive seeds among all seeds, the number of RDS coupons given to participants, chain length (the number of successive RDS recruitment waves), chain size (number of participants from an RDS branch) per RDS chain/seed, the wave at which participants were recruited (measured at wave 0 for all seeds, wave 1 for recruits of seeds, wave 2 for recruits of wave 1 participants, etc.), and the total number of participants in the recruitment chain that the participant is a part of (measured at the participant-level). Further, we asked participants about their main reason for participating in the study and their relationship to the person from whom they received their coupon. Sociodemographic measures included age, race/ethnicity, identification as a person of colour, annual income, education, current employment, whether participants were born in Canada, gender identity/transgender identity (based on birth sex and current gender identity) and sexual identity. These demographic characteristics were identified based on previously assessed literature exploring differential success using RDS among GBM [23]. We also included the financial strain index, which is a comprehensive measure of financial resources. It consists of five items: with responses on a 3-point Likert scale, with ‘‘1’’ denoting ‘‘Not true’’, ‘‘2’’ denoting ‘‘A little true’’, and ‘‘3’’ denoting ‘‘Very true.’’ Answer categories were assigned a value and summed, with higher scores indicating increased financial strain [24].

### Analysis

For each city, we provided crude point estimates for RDS recruitment characteristics. We also illustrate the number of seeds and participants in each respective city and recruitment period (seeds are also considered participants in this study and are included in final sample sizes). Kruskal–Wallis tests were used to compare RDS recruitment characteristics between the three cities (i.e., number of productive seeds, number of direct recruits, participant network size. etc.). We also assessed sociodemographic characteristics by city and provide crude and RDS adjusted estimates (using RDS-II weights) and 95% confidence intervals. RDS-II weights were based on participant’s social network from the question:​ “How many men who have sex with men aged 16 years or older, including trans men, do you know who live or work in the Metro Vancouver/Toronto/Montreal area?” [25]​. Furthermore, we include recruitment homophily estimates for each study site, calculated using RDSAT Version 7.1.46. Homophily is a diagnostic tool that describes the mixing patterns in networks and is used in this analysis to examine participants similarity in a recruiter and recruited participant characteristics, relationship to referrer, and reasons for study participation [26]. Scores range between − 1 (completely recruiting outside one’s group) and + 1 (completely recruiting within one’s group), with a homophily score of 0 indicating that all recruits are formed randomly, without consideration for group characteristic. A homophily score of 0.3 (or − 0.3) was considered as “substantial” in-group (or out-group) recruitment [26]. Statistically significant differences were assessed using 95% confidence intervals and p-values less than 0.05. All analyses were completed using SAS 9.4 (SAS Institutes, Cary, NC).

## Results

### Overview of RDS recruitment and recruitment characteristics

A total of 2,449 participants were recruited: 753 GBM in Vancouver, 517 GBM in Toronto, and 1179 GBM in Montreal. Vancouver recruited 117 initial seeds of which 60.7% were productive (seeds that recruited at least one additional study participant). Toronto recruited 96 seeds of which 55.2% were productive. Montreal accounted for the smallest number of total seeds (n = 27) and had the greatest percentage of productive seeds (77.8%) compared to the other cities. Montreal obtained the largest sample size over the shortest recruitment period across the three cities (493 days). Assessing RDS process indicators, Montreal also had the largest average chain length (Median = 2, Q1-Q3 = 1–7) and chain size (Median = 7, Q1-Q3 = 2–39) per RDS chain per seed. For both chain length (*p* = 0.001) and chain size (*p* = 0.001), we found significant differences between the three cities. Montreal had the greatest number of participants in their recruitment chain (Median = 274, Q1-Q3 = 75–413) compared to Vancouver (Median = 27, Q1-Q3 = 7–57) and Toronto (Median = 23, Q1-Q3 = 5–61); *p* < 0.001. Full results are in Table 1.

**Table 1:** Overview of RDS Recruitment for the Engage Study

	Vancouver		Toronto		Montreal	
**Sample size**	753		517		1179	
**Interview date earliest**	16-Feb-17		16-May-17		07-Feb-17	
**Interview date latest**	31-Jul-19		09-Aug-19		15-Jun-18	
**Time difference in days**	895		815		493	
**# of seeds**	117		96		27	
**# of productive seeds**	71		53		21	
**% of productive seeds among seeds**	60.7%		55.2%		77.8%	
**# of RDS coupons given to the participant**	4313		3096		6753	
	**Median**	**Q1, Q3**	**Median**	**Q1, Q3**	**Median**	**Q1, Q3**
**# of direct recruits per seed**	1	0, 2	1	0, 2	2	1, 3
Chain length per RDS chain/seed*	1	0, 3	1	0, 1.5	2	1, 7
Chain size per RDS chain/seed*	2	1, 6	2	1, 3.5	7	2, 39
**Participant network size**	30	14, 100	37	15, 100	30	15, 80
Wave at which participant was recruited (participant-level)*	2	1, 4	2	1, 4	6	4, 9
Total number of participants in recruitment chain (participant-level)*	17	7, 57	23	5, 61	274	75, 413

*= Kruskal-Wallis test p-value significant at p < 0.05

### Recruitment by seeds and participants

Table 2 highlights seed and participant recruitment by 3-month period in Vancouver, Toronto, and Montreal. Recruitment began in Vancouver in February 2017 with 13 seeds who then recruited 8 participants, and in Toronto, recruitment commenced in April 2017 with 24 seeds who recruited 18 participants in that time period. Additional seeds were purposefully added throughout the recruitment period in both Vancouver and Toronto to promote recruitment numbers and diversity of study populations. By the end of recruitment, Vancouver had a total of 753 participants, 117 of these being seeds. By the end of recruitment in Toronto, there was a total of 517 participants, 96 of these being seeds. In Montreal, during February-March 2017, 86 participants were recruited, with 25 of these being seeds. Other than two additional seeds added during April-June 2017, Montreal did not add any additional seeds after this point due to steady study recruitment. Recruitment in Montreal ended with a total of 1,179 participants with 27 seeds.

**Table 2:** Recruitment in the Engage Study for Seeds and Participants

	Calendar Time											
Vancouver	Feb-Mar 2017	Apr-Jun 2017	Jul-Sep 2017	Oct-Dec 2017	Jan-Mar 2018	Apr-Jun 2018	Jul-Sep 2018	Oct-Dec 2018	Jan-Mar 2019	Apr-Jun 2019	Jul-Aug 2019	Total
**Participants**	8	33	28	50	88	122	66	49	77	71	44	636
**Seed**	13	15	10	23	8	11	8	9	8	10	2	117
**Total**	21	48	38	73	96	133	74	58	85	81	46	753
**Toronto**	**Feb-Mar 2017**	**Apr-Jun 2017**	**Jul-Sep 2017**	**Oct-Dec 2017**	**Jan-Mar 2018**	**Apr-Jun 2018**	**Jul-Sep 2018**	**Oct-Dec 2018**	**Jan-Mar 2019**	**Apr-Jun 2019**	**Jul-Aug 2019**	**Total**
**Participants**	0	18	60	64	48	29	52	37	59	30	24	421
**Seed**	0	24	3	3	5	13	7	16	2	11	12	96
**Total**	0	42	63	67	53	42	59	53	61	41	36	517
**Montreal**	**Feb-Mar 2017**	**Apr-Jun 2017**	**Jul-Sep 2017**	**Oct-Dec 2017**	**Jan-Mar 2018**	**Apr-Jun 2018**	**Jul-Sep 2018**	**Oct-Dec 2018**	**Jan-Mar 2019**	**Apr-Jun 2019**	**Jul-Aug 2019**	**Total**
**Participants**	61	190	240	224	262	175	0	0	0	0	0	1152
**Seed**	25	2	0	0	0	0	0	0	0	0	0	27
**Total**	86	192	240	224	262	175	0	0	0	0	0	1179

### Sociodemographic characteristics of participants by study site

Overall, 45.4% of recruits in Vancouver were under age 30, compared with 50.7% under 30 in Toronto, and Montreal had the lowest proportion (36.4%). Homophily results highlight lower homophily in Toronto for participants recruited under 30 years of age (0.09), compared to Montreal (0.44) and Vancouver (0.40). Conversely, Montreal had the largest proportion of participants who were 45 years and older (29.1%), compared to 24.6% in Vancouver and 21.0% in Toronto. (*p* < 0.001*)*. Montreal also had greater homophily for recruiting participants 45 years of age and older (0.63) compared to (0.53) for Vancouver and (0.50) for Toronto, but homophily for all three sites were substantially high. Across all three cities, the majority of GBM self-identified as having Canadian ethnicity, with 40.0% identifying as Canadian in Vancouver, 34.8% in Toronto, and 55.0% in Montreal (*p* < 0.001). Homophily results for ethnoracial identity reflected these differences and was substantially high in Montreal (0.42) compared to Toronto (0.21) and Vancouver (0.26). Regarding annual income, 61.3% of GBM reported an annual income of less than $30,000 in Vancouver, compared to 57.4% in Toronto, and 66.8% in Montreal. However, homophily measures were similar and low across the three cities, Montreal (-0.02), Toronto, (-0.03) and Vancouver (-0.03). Conversely, Montreal reported the lowest percentage of participants with an annual income $60,000 and greater (7.9%) compared to 13.1% in Vancouver and 10.6% in Toronto (*p* < 0.001). Homophily results for high income participants were similar and relatively high across the three cities, Montreal (0.28), Toronto (0.33), and Vancouver (0.30). Montreal had the highest homophily (0.16) for recruiting participants with annual incomes $30,000–59,000 compared to Toronto (0.10) and Vancouver (0.05), but all values were quite low, indicating limited impact on recruitment. For education, we found that Montreal had the highest proportion of participants who reported a high school or equivalent education (25.4%) compared to Toronto (12.1%) and Vancouver (16.5%) (*p* < 0.001). However, homophily results were similar and low across the three cities: 0.04 in both Montreal and Toronto, and 0.14 in Vancouver, indicating little effect on recruitment. We also found 76.8% of participants in Vancouver reported having a greater than high school education, compared to 77.2% in Toronto and 64.3% in Montreal (*p* < 0.001). Homophily values suggest a moderate impact of education on participant characteristics across all 3 cities, with 0.37 in Montreal, 0.46 in Toronto, and 0.32 in Vancouver. Lastly, we found 62.5% of participants in Vancouver self-reported negative HIV serostatus, compared to 65.4% in Toronto and 73.7% in Montreal (*p* < 0.001). Homophily results reflected these differences, as Montreal had lower homophily for recruiting HIV-negative GBM (0.24) compared to Vancouver (0.54) and Toronto (0.34), which were substantially higher. For GBM who self-identified as living with HIV, Montreal had the lowest proportion of participants (12.7%) compared to Toronto (18.4%) and Vancouver (19.4%). However, homophily results were substantially high across the three cities, Montreal (0.39), Toronto (0.42), and Vancouver (0.41). Financial strain in Vancouver (adjusted median:6) was lower compared to Toronto (adjusted median: 7) and Montreal (adjusted median: 7) (*p* < 0.001). Full sociodemographic results can be found in Table 3.

**Table 3:** Characteristics of Participants in Vancouver, Toronto and Montreal

	Vancouver (n = 753)					Toronto (n = 517)				Montreal (n = 1179)				
		RDS Adjusted					RDS Adjusted				RDS Adjusted			
Variable	Homophily	%	95% CI			Homophily	%	95% CI		Homophily	%	95% CI		Adjusted p-value
**Age**														**< 0.001**
Under 30	0.40	45.4	38.5	52.4		0.09	50.7	41.4	60.0	0.44	36.4	31.5	41.3	
30 to 44	0.42	30.0	23.9	36.2		0.41	28.3	20.9	35.8	0.31	34.6	29.2	39.9	
45 and over	0.53	24.6	18.2	30.9		0.50	21.0	11.4	30.6	0.63	29.1	24.1	34.0	
**Ethnicity**														**< 0.001**
Canadian	0.26	40.0	33.2	46.8		0.21	34.8	25.5	44.1	0.42	55.0	49.6	60.3	
Indigenous	0.11	4.0	0.0	8.0		-1.00	2.2	0.0	5.4	0.13	1.2	0.0	2.6	
European	0.11	14.5	10.4	18.6		-0.01	21.9	14.3	29.4	0.18	14.7	10.9	18.4	
Asian	0.25	22.2	16.4	28.0		0.09	15.7	8.5	22.9	0.16	5.2	2.8	7.5	
African/Caribbean/Black	0.24	1.8	0.0	3.8		0.25	6.7	2.5	10.9	0.13	3.2	1.6	4.8	
Mixed Race	0.05	3.0	0.6	5.4		-1.00	4.1	1.4	6.7	-1.00	1.9	0.9	2.8	
Other	0.26	14.5	9.3	19.7		0.32	14.7	9.3	20.0	0.27	19.0	14.4	23.6	
**Person of colour**														**< 0.001**
No	0.29	69.6	62.7	76.4		-0.01	76.6	70.0	83.2	0.18	87.6	83.9	91.4	
Yes	0.14	30.4	23.6	37.3		0.12	23.4	16.8	30.0	0.13	12.4	8.6	16.1	
**Annual income**														**< 0.001**
Less than $30,000	-0.03	61.3	54.9	67.8		-0.03	57.4	48.0	66.7	-0.02	66.8	62.2	71.5	
$30,000 to $59,999	0.05	25.6	19.7	31.5		0.10	32.0	22.5	41.5	0.16	25.3	21.0	29.6	
$60,000 and over	0.30	13.1	10.0	16.2		0.33	10.6	7.0	14.3	0.28	7.9	5.6	10.1	
**Education**														**< 0.001**
Less than highschool	0.21	6.7	1.9	11.5		-1.00	10.7	2.2	19.2	0.11	10.2	7.0	13.4	
Highschool or equivalent	0.14	16.5	11.9	21.2		0.03	12.1	7.2	17.1	0.04	25.4	20.0	30.8	
Greater than high school	0.32	76.8	70.7	82.9		0.46	77.2	68.2	86.1	0.37	64.3	58.8	69.9	
Table 3**Cont’d: Characteristics of Participants in Vancouver, Toronto and Montreal**														
**Current employment**														**< 0.001**
No	0.16	29.3	23.0	35.5		0.05	47.4	37.9	57.0	0.09	43.4	37.9	48.9	
Yes	0.35	70.7	64.5	77.0		0.53	52.6	43.0	62.1	0.42	56.6	51.1	62.1	
**Born in Canada**														**< 0.001**
No	0.13	42.7	35.9	49.5		0.20	42.6	33.7	51.6	0.30	34.9	29.8	39.9	
Yes	0.38	57.3	50.5	64.1		0.28	57.4	48.4	66.3	0.42	65.1	60.1	70.2	
**Sexual identity**														**0.035**
Gay	0.41	79.6	73.3	86.0		0.39	72.4	63.9	80.9	0.45	75.3	70.4	80.1	
Bisexual	0.10	9.5	5.4	13.6		0.18	13.6	5.8	21.4	0.23	12.7	9.0	16.5	
Other	0.18	10.9	5.3	16.4		0.21	14.0	8.8	19.3	0.25	12.0	8.2	15.7	
**Gender identity**														**< 0.001**
Man	0.49	93.3	88.9	97.8		0.50	92.0	87.2	96.8	0.44	88.4	84.2	92.6	
Trans man	0.19	0.8	0.0	1.7		0.28	1.0	0.2	1.7	0.16	1.5	0.3	2.7	
Genderqueer/Gender non-conforming	0.08	1.3	0.4	2.1		0.04	3.6	0.2	7.0	0.18	2.2	1.0	3.5	
Two spirit	0.15	3.8	0.0	8.1		-1.00	0.6	0.0	1.4	-0.26	4.9	1.4	8.4	
Another identity	-1.00	0.8	0.0	1.6		-1.00	2.8	0.0	6.2	0.03	2.9	0.7	5.2	
**Self-reported HIV status**														**< 0.001**
HIV-Negative	0.54	62.5	55.5	69.5		0.34	65.4	55.3	75.5	0.24	73.7	69.0	78.3	
HIV-Positive	0.41	19.4	13.6	25.2		0.42	18.4	9.8	27.0	0.39	12.7	9.8	15.6	
Unknown	-0.15	18.1	12.3	23.8		-0.38	16.2	7.5	24.9	0.11	13.7	9.6	17.7	
Continuous Variables	Median	(Q1-Q3)	Adjusted Median	Adjusted Q1	Adjusted Q3	Median	(Q1-Q3)	Adjusted Median	Adjusted Q1	Adjusted Q3	Median	(Q1-Q3)	Adjusted Median	
**Financial Strain Sum Score**	6	(5–9)	6	5	9	7	(5–10)	7	5	10	7	(5–9)	7	**< 0.001**

### Reasons for participation and relationship with recruiter

Overall, the most reported reason for participation was being “interested in sexual health and HIV”, with 34.7% identifying this as their primary motive for study participation in Vancouver, 29.8% in Toronto, and 36.1% in Montreal. There were wide variations in homophily results between the three cities for this response (Montreal=-0.03; Toronto = 0.03; Vancouver=-0.18). However, homophily was very low overall, signalling little effect on within group recruitment. Assessing the relationship participants had with their recruiter, participants from Montreal were more likely to indicate they were referred by an acquaintance (28.4%), compared to Toronto (11.2%) and Vancouver (19.0%) (*p* < 0.001). Homophily results for this outcome were mixed (Montreal = 0.09; Toronto = 0.18; Vancouver = 0.16), and homophily was low overall. Full results are in Table 4.

**Table 4:** Reasons for Study participation and Relationship with Recruiter Among Participants in Engage

	Vancouver (n = 753)				Toronto (n = 517)				Montreal (n = 1179)				
		RDS Adjusted				RDS adjusted				RDS Adjusted			
	Homophily	%	95% CI		Homophily	%	95% CI		Homophily	%	95% CI		Adjusted p-value
**Reason for study participation**													**< 0.001**
Interested in sexual health and HIV	-0.18	34.7	27.8	41.5	0.03	29.8	22.4	37.2	-0.03	36.1	30.8	41.5	
Interested in gay men’s issues	0.07	17.3	12.4	22.3	0.09	11.3	6.8	15.8	0.11	16.3	12.9	19.8	
My friend/partner wanted me to participate	0.03	15.7	11.1	20.3	-0.45	21.9	13.2	30.6	-0.02	9.9	7.2	12.6	
Wanted to help the community	0.08	21.5	15.8	27.3	-0.13	30.4	20.9	40.0	0.06	23.7	18.9	28.5	
I am mostly interested in the $50 incentive for participating	0.06	10.6	5.9	15.2	-0.43	5.7	3.3	8.1	0.14	12.7	9.3	16.0	
None of the above	-1.00	0.2	0.0	0.3	-1.00	0.8	0.0	1.9	-1.00	1.3	0.5	2.1	
**Relationship of participant to person who gave voucher**:													
**Partner**													0.246
No	0.20	72.6	66.0	79.1	0.04	68.3	59.7	76.8	0.08	71.2	66.2	76.2	
Yes	0.04	27.4	20.9	34.0	-0.19	31.7	23.2	40.3	0.03	28.8	23.8	33.8	
**Friend**													0.224
No	-0.11	51.9	45.1	58.8	-0.09	47.5	38.2	56.8	-0.001	51.7	46.3	57.0	
Yes	0.26	48.1	41.2	54.9	0.16	52.5	43.2	61.8	0.06	48.3	43.0	53.7	
**Acquaintance**													**< 0.001**
No	0.13	81.0	74.9	87.2	-0.02	88.8	84.7	93.0	0.07	71.6	66.7	76.5	
Yes	-0.16	19.0	12.8	25.1	0.18	11.2	7.0	15.3	0.09	28.4	23.5	33.3	
**Stranger**													0.999
No		100.0	100.0	100.0		100.0	100.0	100.0	0.49	99.8	99.5	100.0	
Yes									0.50	0.2	0.0	0.5	
**Other**													**< 0.001**
No	0.33	84.6	79.4	89.8	-0.001	84.5	77.8	91.1	-0.01	96.3	94.7	98.0	
Yes	0.04	15.4	10.2	20.6	-0.10	15.5	8.9	22.2	0.05	3.7	2.0	5.3	

## Discussion

We examined recruitment characteristics, sociodemographic characteristics of participants, reasons for participation, and the relationship with referrers to better understand differences in Montreal’s greater level of recruitment using RDS compared to Toronto and Vancouver. Participant characteristics and related homophily measures were largely similar for the three cities, however, there were some notable differences in age, income, ethnicity and HIV status, but we did not find significant differences in reasons for participation.

Although previous research has shown that network size is an important predictor of successful RDS recruitment [27], we did not find significant differences in participant network sizes across the three cities. Indeed, it may not be the number of relations but rather the quality of these relationships that may be important for RDS recruitment. As such, more established relationships between participants may have facilitated comfort in distributing RDS coupons, as well as more purposeful coupon distribution to participants who would be eligible and willing to participate. Evidently, there was a large disparity in the number of seeds in each city and impact on study recruitment. Given that Montreal had the fewest number of seeds but the highest proportion of productive seeds, future research could examine the process of seeds identification and selection on RDS recruitment. Furthermore, including qualitative interviews or focus groups with participants when study recruitment begins may bring a nuanced perspective on how recruitment is working and how to overcome potential challenges.

Examining sociodemographic factors, Montreal had the lowest proportion of participants under the age of 30 (36.4% in Montreal vs. 45.4% in Vancouver and 50.7% in Toronto) and the highest proportion of participants aged 45 or older (29.1% in Montreal vs. 24.6% in Vancouver and 21.0% in Toronto) across the three cities. While homophily was high across all three sites (0.50 for Toronto and 0.53 for Vancouver), homophily was greatest in Montreal (0.63) for recruiting participants 45 years or over. Homophily results imply that GBM over 45 years old from Montreal recruited from within their age group 63% of the time, and GBM from Vancouver 53% of the time, and GBM from Toronto 50% of the time. Age results possibly suggest that older recruits with closer social networks were associated with recruitment success [28, 29]. These findings on older GBM echo previous GBM studies which identified that young GBM tend to establish friendships with other young GBM [30]. However, age differences were small compared to the overall difference in recruiting speed in Montreal.

Regarding income, Montreal had the highest proportion of participants with an annual income less than $30,000 and the lowest proportion with an annual income greater than $60,000. We found high homophily for participants with an annual income greater than $60,000 across all three study sites. Our findings are aligned with other research which has found that individuals with similar income levels tend to be connected with each other in social networks [31, 32]. However, we did not find that the higher proportion of lower income participants associated with recruitment, as homophily for income <$30,000 in Montreal was very low (-0.02), indicating very little effect on within-group recruitment. Additionally, examining the financial strain index, which provides a more holistic measure of financial resources, we found participants in Vancouver had lower financial strain than participants in Toronto and Montreal, which may reflect the greater proportion of participants in Vancouver with an annual income of $60,000 or more compared to Toronto and Montreal.

We also cannot conclude that the financial incentive was the cause for RDS success in Montreal, as the majority of participants reported “interest in sexual health and HIV” as the main reason for their participation (36.1%), which was similar to results from Toronto and Vancouver. Furthermore, only 12.7% of participants from Montreal indicated their main interest in study participation was the financial incentive compared to 5.7% in Toronto and 10.6% in Vancouver. Homophily for the financial incentive in Montreal was also low (0.14), while homophily in Toronto was − 0.43 and in Vancouver, 0.06. To further understand the importance and impact of participant motivation, we propose expanding questions on motivations to include perceptions and experiences with the organization leading the study, having a better sense of participant ease and interest in engaging in research.

We also found that Montreal had the greatest proportion of participants to self-identify their ethnicity as Canadian (55.0%), and homophily results reflected these differences, as homophily for Canadian ethnicity was highest in Montreal (0.42), whereas other sites homophily were not substantial. These ethnic identity differences are consistent with Montreal’s population demographics, where according to data from the Canadian Census, the percentage of residents who identify their racial origin as Canadian is higher compared to Toronto and Vancouver [33]. However, our findings are incongruent with previous research, which found that Black GBM had greater racial homophily compared to white and Latino GBM [34]. In our analyses, we did not find that African/Caribbean/Black participants had significant homophily. Interestingly, we found a homophily of -1 (recruited completely outside of one’s group) for Indigenous participants in Toronto, and mixed-race participants in Toronto and Montreal.

Finally, Montreal had the lowest proportion of participants who self-reported as HIV-positive and the highest proportion of participants who were HIV-negative. Homophily for HIV-negative status was substantially lower in Montreal (0.24) compared to the other sites, but we found homophily for recruiting GBM living with HIV to be substantially high across the three cities. These findings are in contrast with previous homophily research, which found very low rates of homophily among GBM living with HIV and negative scores of homophily for HIV-negative GBM [35]. Overall, we suspect that homophily for demographic characteristics which people build their social networks on (e.g. race, age, ethnicity) may be high, but we hypothesize that homophily dilutes over time with several waves of recruitment and as recruiters become more heterogenous.

Differences in the success of RDS may also be associated with geography and culture. Montreal has a distinct French-speaking culture with a significantly different history and identity compared to most large Canadian cities. Of note, Montreal has one of the largest gay villages (measured in geographical size) in North America [36]. Correspondingly, results from a national online survey of GBM in Canada, conducted in 2019, found men in Quebec report the highest satisfaction for their connection with other GBM (76.8% in Quebec vs. 56.4% in BC and 61.0% in Ontario), the highest satisfaction for physical spaces to connect with other GBM (66.6% in Quebec vs. 45.3% in BC and 49.7% in Ontario) and highest satisfaction for online spaces to connect with other GBM (71.4% in Quebec vs. 65.0% in BC and 67.7% in Ontario) [37]. The impacts of geography and social-cultural connectedness warrants further exploration on associations with RDS methodology and recruitment. Other considerations which may have affected recruitment is the number of GBM residing in each city and the geographic location of study sites (Toronto had a university-based site while Vancouver and Montreal had community-office based sites). Apart from this difference, public transportation in all three study sites was very accessibly, with a direct bus stop within one block of the sites. Additionally, staff training was the same for all sites. However, study hours varied by location, with Toronto having shorter but later study hours from 3pm-9pm (5 days a week, including Saturday), Vancouver from 11am-7pm (5 days a week, including Saturday), and Montreal from 10am-6pm (5 days a week, including Saturdays). Additionally, Montreal was the only site to offer online appointment booking, which may have also contributed to enrolment success. Lastly, Montreal was the only site to have a store-front, meaning participants could directly enter the study offices without interacting with other individuals (the Vancouver site was an office space within a medical building and the Toronto site was within a university building). The Montreal site was also previously home to a community-testing site, which was well-known in the LGBT2Q community, and we believe this familiarity with the site as well as direct access impacted recruitment.

Our study findings are similar to previous research from Murill et al. (2016), which found significant differences in seed numbers, productivity of seeds and recruits in their three-city RDS study among Black and Latino GBM in New York, Los Angeles and Philadelphia. The authors suggest that differences in population characteristics may reflect each city, but suggest that differences may be due to inevitable data collection and implementation at each site [23]. Our study had minor differences in study implementation regarding participant follow up, which each site undertook based on their own recruitment progress. While Montreal and Toronto systematically followed up with study participants throughout their recruitment period, Vancouver only opted for one round of follow-up from participants and instead chose to add more seeds earlier on; this decision was based on previous experience from the Momentum Health Study [38–40]. Flexibility in RDS protocol is recommended for multi-city studies, however, examining the impact of differences such as added seeds or follow-up procedures is an important consideration for RDS. Another important consideration for Vancouver was that Engage was built upon the success of the Momentum Health Study, which ran for 5 years prior to Engage recruitment and used RDS methodology. Thus, to reach new members of the community, prior participants were unable to be seeds in Engage. This proved challenging, as some of the most well-connected members of the community had already participated in the original study as seeds. Additionally, some participants who were eager to participate in the current study, having previously participated in Momentum and having been successful recruiters, had to wait until they had received a coupon organically, which did not always happen. However, recruitment in Vancouver largely proceeded at the same pace and with the same number of seeds as it did in Momentum. In addition to Momentum in Vancouver, Vancouver has a history of engaging GBM in large community-based studies such as Mancount [41] and the Sex Now Survey [42]. The same could also be said for Toronto, which also has a history of engaging GBM in large community-based studies such as the iCruise study [43], Imagine Men’s Health Study [44], Mbawana Black Men’s study [45], and Pride Toronto [46]. In contrast to Vancouver and Toronto, there has been a dearth of GBM research in Montreal, and it is possible that this also affected recruitment success. Assessing the influence of research fatigue in future RDS studies is warranted, and asking about previous experience with research would also be helpful for future RDS studies.

This study has a number of strengths and limitations. First, our study only has data for those who chose to participate in our study. Ideally, we would have liked to have information on potential participants who were invited but declined participation, which was not possible. Furthermore, RDS recruitment is based on social networks, and GBM who are not connected with the lesbian, gay, bisexual, transgender, two-spirit, and queer (LGBT2Q) communities, or those who are isolated may be underrepresented. Although we attempt to address this limitation by identifying hidden subpopulations and recruiting purposeful and diverse seeds, individuals who are not “out” and unknown to researchers may have led to some selection bias and confounding. A challenge with RDS is choosing seeds who are well connected with other individuals in the population of interest, potentially excluding other hidden populations. However, starting with seeds who are less connected and harder to find may limit the RDS process of chain-referral sampling since this involves peer recruitment. Finally, a general limitation of RDS is its generalizability to the actual population of interest, which also applies to the study presented here.

Despite these limitations, a major strength of this study is that we had three sites which allowed us to compare RDS characteristics between them and used the same recruitment strategies and study procedures in each city. Overall, RDS methodology is an economical and beneficial methodology for reaching hard-to-reach populations who may not be accessible at traditional venues, and who may have not participated in research on their own.

## Conclusion

Our study examined multiple factors to explain differences in recruitment success for Engage in Montreal, Toronto, and Vancouver. While we found some differences in participant characteristics and homophily, these differences were minimal and likely did not have a major impact on recruitment in Montreal. Notably, we found no differences in reported reasons for study recruitment across sites. While we support the use of RDS as a recruitment strategy for studies of GBM in different settings, our study underlines the fact that success of implementation may vary by unknown factors, and that researchers should be proactive and flexible to account for variability. Further research is needed to understand how and why RDS works well or does not work in the many settings in which it is being used.

## Data Availability

The datasets generated and/or analysed during the current study are not publicly available due to privacy concerns for this ongoing cohort study. Deidentified participant data used in this analysis are stored at the BC CfE. For information regarding these databases, and related access, please contact David Moore, a principal investigator on the Engage Study.

## List of abbreviations

RDS: Respondent-driven sampling
GBM: Gay, Bisexual and other Men who have sex with men
CASI: computer-assisted self-interview
CAD: Canadian Dollars
LGBT2Q: lesbian, gay, bisexual, transgender, two-spirit and queer
